# Foodborne Diseases

**DOI:** 10.3201/eid1407.080346

**Published:** 2008-07

**Authors:** Ynes R. Ortega

**Affiliations:** * University of Georgia, Griffin, Georgia, USA

**Keywords:** foodborne diseases, bacterial pathogens, viral pathogens, book review

As the title suggests, this book covers relevant topics on foodborne diseases. Most chapters are descriptive, updated, and, given likely page-length limitations, concise. Of 21 chapters, 6 cover selected foodborne bacterial pathogens: *Escherichia coli, Listeria monocytogenes*, *Clostridium botulinum* and *C. perfringens, Yersinia enterocolitica* and *Y. pseudotuberculosis,* pathogenic *Vibrio* spp., and *Enterococcus* spp.

Two chapters discuss viral pathogens such as hepatitis and gastroenteric viruses. Four chapters focus on parasites: *Cryptosporidium* spp., *Cyclospora* spp*., Giardia* spp., and *Toxoplasma gondii.* Other chapters address aflatoxins; scombroid fish poisoning; food management, including hazard analysis and critical control point programs; antimicrobial agents in food-animal production; alternatives to antimicrobial drugs; and microbial risk assessment. Additional chapters also review new trends for control of foodborne pathogens (food irradiation and other sanitation procedures) and molecular techniques for detecting and identifying foodborne pathogens and their toxins. The last chapter considers future directions of food safety.

One limitation of the book is the lack of thorough discussion of other relevant foodborne pathogens, such as *Salmonella* spp., *Shigella* spp., *Campylobacter* spp., *Bacillus* spp., *Staphylococcus* spp., *Enterobacter sakazakii*, and *Aeromonas* spp. These bacteria are important foodborne pathogens worldwide, and although they are briefly mentioned in other chapters, much more consideration is warranted (*1*,*2*).

For example, in 2006, in the United States, 5,712 cases of *Campylobacter* infection and 6,655 cases of *Salmonella* infection were documented. *Campylobacter* spp. are the most frequently diagnosed causes of gastroenteritis in the United States, and ≈80% of cases are foodborne. Recent well-publicized foodborne outbreaks in the United States have been associated not only with *E. coli* O157:H7, but with *Salmonella* spp. as well. A multistate outbreak of *S.* Typhimurium infections associated with tomatoes accounted for 14% of the cases in 2006. *S.* Newport accounted for 9.2% of the cases. In 2007, >400 cases of *S.* Tennessee infection were attributed to consumption of peanut butter. *E. sakazakii* has caused fatal infections in neonates who were fed contaminated infant formula; this pathogen presents particular challenges to the food industry. In addition, no mention was made of helmintic infections, which also can be associated with foods.

If page limitations were an issue, the 4 chapters dedicated to parasitic infections could have been condensed to 2, and bacterial pathogens could have been emphasized. This would have been doable because 3 of the 4 parasite chapters were written by the same senior author in collaboration with others.

Overall, several relevant topics on foodborne diseases are sufficiently described in this book, and credit should be given to the chapter contributors who provided adequate information on their respective topics. This is a very good reference book for health departments, the food industry, and academia.
